# CGAT: Cell Graph ATtention Network for Grading of Pancreatic Disease Histology Images

**DOI:** 10.3389/fimmu.2021.727610

**Published:** 2021-09-29

**Authors:** Mayank Baranwal, Santhoshi Krishnan, Morgan Oneka, Timothy Frankel, Arvind Rao

**Affiliations:** ^1^ Division of Data & Decision Sciences, Tata Consultancy Services Research, Mumbai, India; ^2^ Department of Systems and Control Engineering, Indian Institute of Technology, Bombay, India; ^3^ Department of Electrical & Computer Engineering, Rice University, Houston, TX, United States; ^4^ Department of Computational Medicine and Bioinformatics, University of Michigan, Ann Arbor, MI, United States; ^5^ Department of Surgery, University of Michigan, Ann Arbor, MI, United States; ^6^ Department of Biostatistics, University of Michigan, Ann Arbor, MI, United States; ^7^ Department of Radiation Oncology, University of Michigan, Ann Arbor, MI, United States; ^8^ Department of Biomedical Engineering, University of Michigan, Ann Arbor, MI, United States

**Keywords:** PDAC (pancreatic ductal adenocarcinoma), cell-graph, spatial method, pancreas, attention network, chronic pancreatitis, graph convolutional network (GCN)

## Abstract

Early detection of Pancreatic Ductal Adenocarcinoma (PDAC), one of the most aggressive malignancies of the pancreas, is crucial to avoid metastatic spread to other body regions. Detection of pancreatic cancer is typically carried out by assessing the distribution and arrangement of tumor and immune cells in histology images. This is further complicated due to morphological similarities with chronic pancreatitis (CP), and the co-occurrence of precursor lesions in the same tissue. Most of the current automated methods for grading pancreatic cancers rely on extensive feature engineering involving accurate identification of cell features or utilising single number spatially informed indices for grading purposes. Moreover, sophisticated methods involving black-box approaches, such as neural networks, do not offer insights into the model’s ability to accurately identify the correct disease grade. In this paper, we develop a novel cell-graph based Cell-Graph Attention (CGAT) network for the precise classification of pancreatic cancer and its precursors from multiplexed immunofluorescence histology images into the six different types of pancreatic diseases. The issue of class imbalance is addressed through bootstrapping multiple CGAT-nets, while the self-attention mechanism facilitates visualization of cell-cell features that are likely responsible for the predictive capabilities of the model. It is also shown that the model significantly outperforms the decision tree classifiers built using spatially informed metric, such as the Morisita-Horn (MH) indices.

## 1 Introduction

In recent years, there has been an increase in the incidence of pancreatic cancers cases (1). Though there are various forms of exocrine and endocrine tumors such as primary pancreatic lymphoma manifest in the pancreas, Pancreatic Ductal Adenocarcinoma (PDAC) accounts for more than 90% of diagnosed malignancies (2). PDAC is an extremely aggressive malignancy of the pancreas, with a reported overall 5-year survival rate of just 10.8% (3). As with other cancers, there have been precursor lesions that have been identified and associated with sequential progression to PDAC, with the most important being intraductal papillary mucinous neoplasm (IPMN), pancreatic intraductal neoplasia (PanIN), and mucinous cystic neoplasm (MCN), all of which have been well-documented in recent years (4, 5). It is possible for MCN and IPMN to be concomitant with PDAC, with both histologies being present in a patient (6). Additionally, it has been observed that a dense inflammatory mass formation is present in around 30% of CP diagnoses, mimicking the appearance of PDAC, posing additional challenges in differential diagnosis (7).

Currently, qualitative visual analysis of tissue biopsy is the prevailing methodology used by pathologists, where diagnosis is made based on visual markers such as tissue morphology and potential cell phenotype distribution. This visually driven approach has been marred with subjectivity, with very low inter-observer agreements in many cases (8, 9). Moreover, though Hematoxylin and Eosin (H&E) is the most widely used staining paradigm, the emergence of molecular and antigenic-based staining paradigms, such as multiplexed immuno-florescence and CODEX had allowed for the identification of more than 20 antigens on the cell’s surface, enabling richer tissue information to be available (10, 11). It is possible to now characterize multiple sub types of different cell phenotypes, of which immune cells are of great interest. Multiple studies have depicted the differences in the interplay between the different immune populations with a positional component being a key prognostic factor in many cancers, including PDAC (12). Quantitative methods such as the Morisita-Horn index and Shannon Entropy that take into consideration the spatial arrangement of single or multiples cell phenotypes, are being increasingly adopted to quantify cellular organization in the tumor environment tissue (13, 14). Though these methods offer some spatially aware intuition about the image in the overall tissue region, the resultant single number metrics does little to capture the richness in disease heterogeneity. Thus, it would be ideal that the full space of spatial information be leveraged to give insight into the different patterns in tumor and immune engagement in different pancreatic diseases. The identification of such differing spatial patterns can assist in screening patients at risk of PDAC, thus allowing for rigorous treatment planning or resection.

In recent years, a graph-theoretic approach to modeling cellular interactions has been extensively explored. For instance (15–17), propose the notion of using “cell-graphs”, where cellular organization modeled using graph theory concepts can be utilized to understand functional relationships between cells exhibiting similar and different phenotypes. Consequently, the evolution of cancer can be effectively modeled as a graph evolution process (18). Given the spatial distribution of tumor and immune cells in a tissue sample, graph-theoretic methods appear as natural solutions to capture the diversity in distributions; however, the classical graph-based methods do not scale well with the number of cells in a sample. In the recent years, graph neural networks (GNNs) (19–21) and their variants have emerged as viable alternatives that can capture the interdependence of nodes within a graph (or cells within a tissue sample) *via* message passing between the nodes of the graph. The paradigm shift from employing classical methods to adopting deep learning methods, such as the graph convolutional networks (GCNs), is particularly fueled by the recent advances in network architectures, optimization algorithms, and their parallelizable implementation. Zhou et al. (22) recently proposed a cell graph convolutional network that uses the basic notion of cell graph coupled with the graph representation capabilities of GCNs for colorectal tumor grading. Their approach to construct cell graph is based on accounting for both cell-level information and the overall tissue micro-architecture. The authors employ extensive feature engineering for accurately delineating the boundaries of each nucleus *via* CIA-Net (23), followed by obtaining representative nuclei using farthest point sampling (FPS) algorithm. The authors identify a set of seventeen nuclear descriptors for their representative nuclei. More recently, the authors in (24) have combined GCN with gene expression profiles for classifying cancer types. Their approach involves designing four GCNs based on co-expression graph, co-expression+singleton graph, protein-protein interaction (PPI) graph, and PPI+singleton graph. Feature design and extraction is extremely critical to the success of both of the above GCN-based approaches, which also limits the applicability of these approaches to scenarios where accessibility to high-fidelity features is difficult.

In this paper, we propose a cell-graph based method for the classification of point patterns derived from mIF-stained histopathology images belonging to six different cohorts of pancreatic diseases. Instead of focusing on extensive feature engineering, we work with cell-graphs consisting of only one feature per node. A modified GCN architecture, comprising of a novel self-attention mechanism, is shown to achieve excellent performance for pairwise classification tasks. We refer to this new GCN architecture as the Cell-Graph ATtention (CGAT) network. The pairwise classifiers are subsequently bootstrapped to build a multi-class classification network, where an input image is predicted to belong to any one of the six different cohorts of pancreatic diseases. The key contributions of this work can be summarized as:

**Construction of cell-graphs**: Unlike existing methods on tumor grading and classification that employ extensive feature engineering, such as evaluation of mean nuclei intensity, GLCM of dissimilarity, GLCM of homogeneity, solidity and orientation, the proposed CGAT network is provided with an input image where only positions and labels (Epithelial, Cytotoxic Lymphocyte and Regulatory-T) of nuclei are known. The position information is used to construct edges of the associated cell-graph based on pairwise Euclidean distances using the *k*-nearest neighbors (kNN) graph algorithm (25). The nuclei feature, i.e., the labels of the nuclei are embedded into the CGAT framework using an embedding layer (26).

**Self-attention mechanism**: The proposed CGAT network incorporates a novel self-attention mechanism (27) at its output in order to facilitate further interactions between the inputs (nodes) of the graph. The self-attention mechanism assigns scores/weights to different node embeddings. The large weighted nodes are likely to contribute more towards the model prediction.

**Multi-class consensus classifier**: Class imbalance across the six different cohorts makes it extremely challenging to be able to train a single classifier for accurately predicting the correct disease type. We alleviate this issue by bootstrapping multiple pairwise classifiers, each trained to accurately distinguish between two different disease types.

**Performance on imbalanced datasets**: Spatially informed metrics, such as the Morisita-Horn (MH) dissimilarity indices, indicate that there is significant overlap between histopathological images between two different classes. Despite the overlap and underlying class-imbalance in data acquisition, our CGAT network performs significantly better on the hold-out validation set as compared to decision tree classifiers trained using the MH indices. We thereby conjecture that CGAT is able to pick on several spatial features without having to explicitly design those features.

The rest of the paper is organized as follows. First, we provide a brief description of the data used as input in our framework. The architectural details of the proposed pairwise CGAT network for any two of the six groups in the study is explained in greater detail. Then, the extension of the pairwise classifiers for each disease pair for multi-class classification problems are discussed subsequently. This is followed by a presentation of the results obtained from our framework. Finally, we briefly discuss the biological significance of the results obtained from our classification framework.

## 2 Materials and Methods

### 2.1 Dataset Preparation

The study cohort consisted of 388 point pattern representations obtained from multiplexed immunofluorescence (mIF) image cellular data belonging to six different pancreatic disease groups, including pancreatic cancer and non-malignant diseases. These images were obtained from patients at the University of Michigan Pancreatic Cancer Clinic who had undergone surgical resection for various pancreatic diseases, and was done in accordance and approval of the University of Michigan Institutional Review Board.The six pathologies represented in this study were, namely, Chronic Pancreatitis (CP), Pancreatic intraepithelial neoplasia (PanIN), Mucinous cystic neoplasm (MCN), Intraductal Papillary Mucinous Neoplasm (IPMN), IPMN associated cancers (Special Dx IPMN), and traditional Pancreatic Ductal Adenocarcinoma (PDAC). A point pattern representation is obtained when each cell identified is represented by a point on a two-dimensional grid, with the cell location determined by the center of the cell. Out of the 388 image point representations available, 56 were identified as CP, 41 as PanIN, 21 as MCN, 89 as IPMN, 38 as Special Dx IPMN and 143 as PDAC.

For the identification of phenotypes, multiplexed immunofluorescent staining was done on a tissue micro-array composed of 0.6mm cores taken from Formalin-fixed Paraffin-embedded (FFPE) tissue blocks, as explained in our previous work (28). In this process, slides underwent serial rounds of antigen retrieval, followed by primary and secondary antibody staining. DAPI nuclear staining was performed for to identify and segment nuclei and assign spatial locations to every cell present. Nuclear stain phenotyping was done using antibodies for the identification of phenotypes, including CD3, CD8, pancytokeratin, and FoxP3 expression. A subset of the mIF images representative of each cohort is included in the Supplementary Material (see **Supplementary Figure 2**). Of the available phenotypes, 3 were of interest to us as advised by the physician: the Epithelial cell, the immunosuppressive Regulatory T-cell (Treg), and the immunoreactive Cytotoxic Lymphocytes (CTL). An epithelial cell was considered to be any cell expressing Pancytokeratin, a Treg cell was any cell expressing FoxP3, and a CTL was identified as one expressing CD3 and CD8, identified through mIF staining and imaging procedures. Note that we do not strongly claim that only these cell types are sufficient for the characterization of these diseases, but rather that they are the cells that the proposed CGAT framework examines as a first pass. These cell sets are theoretically known to interact with each other in a biologically meaningful way. In future, we aim to expand the types of cells that we query in the microenvironment. All phenotyping and processing of mIF images was done on AKOYA Biosciences’ Inform Software. Additional clinical and demographic information is presented in **Table 1**.

**Table 1:** A summary of clinical characteristics of the patient cohort.

Characteristics		CP	PDAC	IPMN	MCN	PanIN	IPMN-associated PDAC
Number of patients		N = 34	N =71	N = 70	N = 16	N = 29	N = 8
Median age at surgery (years)		50	64	64	44	63	NA
BMI (Mean ± SD)		27.16 ± 6.16	28.5 ± 5.61	28.4 ± 7.05	34.05 ± 9.07	23.48 ± 4.56	NA
Smoking Status	Yes	13	33	15	2	5	NA
	No	4	26	12	11	6	NA
	Unknown	17	12	43	3	18	NA
Sex	Male	13	31	15	0	5	NA
	Female	6	29	13	13	7	NA

Missing values were excluded when computing summary statistics in each category.

### 2.2 Classification

#### 2.2.1 Pairwise Classification

**Figure 1:**
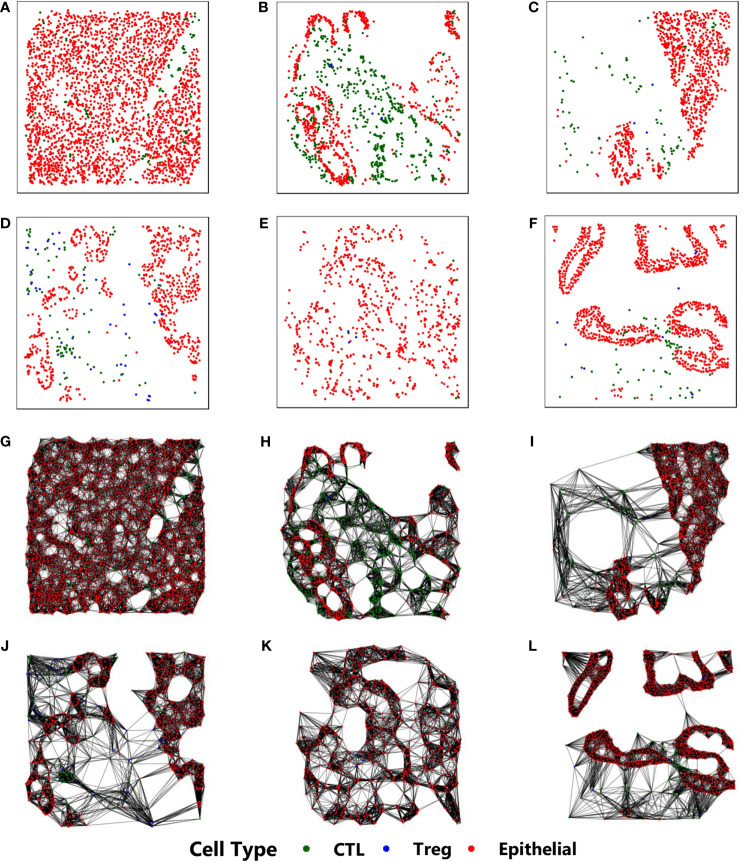
Construction of *k*-NN graphs from point pattern data derived from mIF pathological images from six different classes: **(A)** CP, **(B)** IPMN, **(C)** MCN, **(D)** PanIN, **(E)** PDAC, **(F)** IPMN-associated PDAC. Their corresponding *k*-NN graphs are shown in **(G–L)**, respectively. In all images, the red, blue and green cells correspond to epithelial, cytotoxic lymphocytes, and t-regulatory cells, respectively.

Our approach is based on constructing a *k*-NN (*k*-nearest neighbor) (29) graph from the stained image. The stained image data consists of 2D-coordinates of cell positions, along with the corresponding cell types. Each cell is identified to be one of the three types - (a) Epithelial, (b) Regulatory T (Treg), and (c) Cytotoxic Lymphocyte (CTL). The cell positions are used to construct the *k*-NN graph, while the cell type reflects the property of each cell and is the only physiological feature to be considered as an input to the proposed CGAT architecture. This is in sharp contrast to existing methods that rely on extensive feature engineering, requiring a lot of morphological and physiological features for imparting predictive capabilities (22, 30). **Figure 1** shows sample *k*-NN graphs from the six different classes.

**Figure 2:**
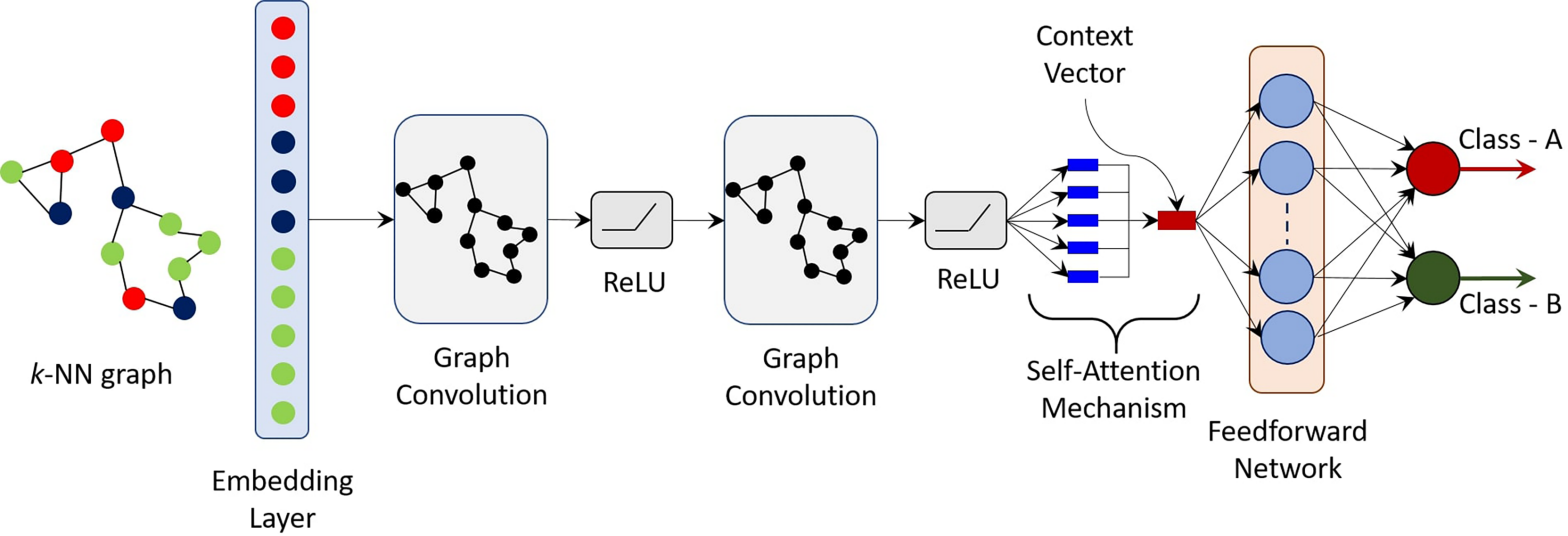
Schematic of the proposed cellular graph attention network (CGAT). Based on the geometrical coordinates of the epithelial and immune cells, a *k*-NN graph is constructed and passed through an embedding layer, which converts cell types labels to a d-dimensional vector. The output of the embedding layer is passed through a GCN with self-attention mechanism. The context vector is finally fed to a simple feed forward network, which maps the input graph to one of the two classes. For the multi-class classifier, 15 such pre-trained pairwise classifiers (one for each combination of classes) are bootstrapped in a rule-based manner.

Every *k*-NN graph defines the corresponding binary graph adjacency matrix, *A*, with *A_i,j_* = 1 if the *j* ^th^-cell is a neighbor (connected) to the *i* ^th^-cell in the *k*-NN graph. In order to employ cell types for learning to classify disease types, a trainable embedding layer is used which converts symbolic cell type labels to a real vector of a specified dimension *d*. Thus for a graph with *N* vertices, the input feature *X*_0_ ∈ℝ*^N^* ^×^ *^d^*. While the proposed work uses onlythree different cell types for disease class prediction, the CGAT framework is quite general and can accommodate any number of cell types by appropriately modifying the maximum size of the dictionary of embeddings in the embedding layer. An *l*-layer GCNupdates the vertex embeddings using the following update rule (20):


(1) $$X_{t+1}=\sigma (\tilde{A}X_{t}W_{t}),\text{for all }t\in \{0,1,\ldots ,l-1\},$$

where $\tilde{A}={\hat{D}}^{-\frac{1}{2}}\hat{A}{\hat{D}}^{-\frac{1}{2}}$ denotes the normalized adjacency matrix with $\hat{A}=A+\text{I}$ and $\hat{D}$ being the degree matrix of $\hat{A}$ . *W_t_* ∈ℝ*^d^* ^×^ *^d^* denotes the weight matrix of the *t* ^th^ layer and is a trainable parameter. *σ* is a nonlinear activation function, such as, rectified linear unit (ReLU) or hyperbolic tangent (tanh). A key component of the proposed CGAT architecture is its novel self-attention mechanism. An attention model helps with focusing on specific parts of the input rather than using all available information to compute the neural response (27). Since its inception, attention models have been used extensively in language-to-language translation, speech recognition and image captioning. The self-attention mechanism of the CGAT takes final node embeddings from GCN as its input and trained to identify vertices (cells) relevant for the prediction task. The self-attention mechanism is specified by the following set of rules:


(2) $$\begin{array}{l} \alpha =\text{tanh}(X_{l})w\\ \;[p]i=\frac{e^{\alpha_{i}}}{\Sigma_{j=1}^{N}e^{\alpha_{j}}}\cdot \text{ for all }i\in \{1,2\ldots ,N\},\\ s=X_{l}^{T}p, \end{array}$$

where *s* ∈ ℝ*^d^* is the resulting *d*-dimensional embedding vector. The parameter *w* ∈ ℝ*^d^* is a trainable parameter, while [*p*] ∈ ℝ*^N^* represents the relative importance of each vertex embedding towards the classification task. It is important to note that the proposed self-attention mechanism converts an *N* × *d*-dimensional embedding to an equivalent *d*-dimensional embedding. Thus, graphs (or stained images) of varying sizes can be easily accommodated as the final context vector *s* is simply *d*-dimensional and independent of the number of cells *N*. Moreover, sizes of the trainable weight parameters are also independent of *N*. Finally, a simple feed forward network (FFN) is used to produce a two-dimensional output vector - one for each target class. The weight matrix in the FFN are learned in an end-to-end manner. A final SoftMax layer is applied to produce a probability, one for each of the possible classes. **Figure 2** shows the schematic of the proposed CGAT architecture for the pairwise classification of pancreatic cancer and precursor types. CGAT assigns to each graph in the dataset, a different embedding, and subsequently a different context vector, which can then be used to predict the correct disease class.

#### 2.2.2 Multi-Class Classification

The anonymized dataset is highly imbalanced in its classes. For instance, there are significantly more number of examples of PDAC (*N=143*) than MCN (*N=21*). Consequently, training an end-to-end multi-class CGAT classifier biases the model towards PDAC. At the same time, a pairwise classifier aimed to distinguish a given pancreatic disease type from the rest of the classes in a one-*vs*-rest classification manner suffers from a similar class imbalance issue. Thus, we build a sequence of pairwise classifiers, each trained to distinguish between two different classes. For six unique classes, we have a pair of 15 unique pairwise classifiers that are bootstrapped to build a multi-class classifier. Note that bootstrapping does not entail training any new classifier, however, it is built upon the pretrained pairwise classifiers sequenced in a rule-based manner. For instance, given the superior performance of pairwise classifiers involving PDAC and CP classes, a consensus based-approach is built to first identify if the input belongs to one of these two classes. We similarly leverage high-performing pairwise classifiers in case the input belongs to any other class.

#### 2.2.3 Model Implementation

All models are implemented in Python 3.6.5 using PyTorch (31) on an Intel i7-7700HQ CPU with 2.8GHz x64-based processor and an NVIDIA GeForce GTX 1060 GPU. The hyperparameters of our CGAT implementation are as follows: optimizer: Adam optimizer (32) with learning rate *λ* = 10^-3^; loss function: cross-entropy; number of epochs = 100; embedding dimension *d* = 30; number of GCN layers *l* = 2; and number of nearest neighbors in *k*-NN graph *k* = 20. The choice of $k=20$ is motivated by the need to strike an optimal balance between a connected only and a complete graph. For the purpose of creating cell graphs and employing a graph neural network architecture, it is desirable to work with graphs that are connected, i.e., there exists a path (a set of edges) between any two nodes in the graph. This facilitates efficient message passing through the cell graph during the aggregation phase of GCN operation. At the same time, it is not desirable to work with complete graphs, i.e., all nodes are connected through direct edges between them, as the node or region-specific properties would not be sufficiently expressed. The code for the implementation of CGAT framework is available on request.

### 2.3 Model Interpretation Using the Giotto framework

We also wanted to explore neighborhood relationships of the cells which were identified as having high “self-attention weights” in the images for the binary classifier. To achieve this, we perform cell-pair enrichment analysis using Giotto (33). For each sample, a cell neighbor graph is created, in which each node represents a cell, and each node is connected to the cell’s three nearest neighbors. A simulation distribution is then created by shuffling the nodes’ cell type labels, while keeping the graph topology the same. For a cell pair of interest, the number of edges between nodes of those two cell types in the original sample is compared to the distribution of edges in the simulations. If the number of edges is significantly higher compared to the simulation distribution, that cell pair is considered to be “enriched”, where cells of those two types are neighbors more than would be expected at random. In contrast, if the number of edges is significantly lower compared to the simulation distribution, that cell pair is “depleted”, where the cells of those two types are neighbors less frequently than would be expected at random. This is done both for cells of the same phenotype as well as cells belonging to different phenotypes. Implementation of the framework using the Giotto package, and other associated analyses were implemented in R [R Core Team (2013)] (33).

## 3 Results

Of the 388 immunofluorescent stained images that belong to one of the six pancreatic disease types, nearly 80% of the data is selected randomly for training while the remaining 20% is held out for cross-validation study. The held-out samples are kept separate and the model performances are evaluated on the test set at the end of the training process. In essence, the train/test split includes 44/12 samples for CP, 71/18 for IPMN, 16/5 for MCN, 33/8 for PanIN, 113/30 for PDAC and 30/8 for IPMN-associated PDAC. The training process is further subjected to a 5-fold cross-validation study. Unlike the standard train/test split, a *k*-fold cross-validation study results in a less biased or less optimistic estimate of the model. In a *k*-fold cross validation study, the training data is split randomly into *k* groups of equal sizes. Subsequently, each unique group is considered in an iterative manner as a hold-out or test set, while the model is trained on the remaining *k* - 1 groups. At the end of each training phase, the trained model is discarded while the evaluation scores are retained. Finally, the performance of the model is summarized using the sample of model evaluation scores on each unique group. For validation purposes, the classification results from our cell-graph attention network classification paradigm was compared with those generated using another spatially informed metric: the Morisita-Horn index (34, 35). This metric has shown to be prognostic in many diseases, including breast cancer (13). A brief description of this method is given in the supplementary section of this paper.

It can be observed that there is significant class imbalance, with MCN having only 16 training samples. A 5-fold cross-validation study further reduces the number of training examples to about 12 or 13 for each unique group. Consequently, the models are going to be biased towards predicting other classes. Despite the absence of sufficient number of examples to train a neural network model, the proposed CGAT framework performs appreciably well.

**Table 2** shows the AUC, precision and recall performances of 15 pairwise classifiers for the aforementioned 5-fold cross-validation study on the held-out test set. For every pairwise classifier, the corresponding row and column entries indicate classes 1 (positive) and 0 (negative), respectively. Recall that a large value of AUC (area under curve) implies that the model has a good measure of separability. On the other hand, precision and recall capture the positive predictive value (PPV) and sensitivity of a model. In order to fully evaluate the effectiveness of the model, both precision and recall scores must be examined, since improving precision typically reduces recall and vice versa. Despite the significant class imbalance, it can be seen in **Table 2** that most of the pairwise classifiers perform appreciably well, with PDAC being the most easily distinguishable class. Likewise, the performance of pairwise classifiers in distinguishing CP is significantly high. On the other hand, it appears generally difficult to distinguish classes, such as, MCN and PanIN, both having relatively fewer training examples to work with. We leverage the superior performance of PDAC and CP classifiers in building a bootstrapped multi-class classifier.

**Table 2:** Classification metrics for the 15 pairwise CGAT classifiers from every point pattern set from each disease group.

Groups	Scores	IPMN	MCN	PanIN	PDAC	IPMN-associated PDAC
	*AUC*	0.65 ± 0.04	0.83 ± 0.07	0.75 ± 0.16	0.93 ± 0.01	0.84 ± 0.13
**CP**	*Precision*	0.61 ± 0.05	0.80 ± 0.40	0.83 ± 0.26	0.92 ± 0.05	0.76 ± 0.15
	*Recall*	0.99 ± 0.02	0.42 ± 0.29	0.40 ± 0.19	0.89 ± 0.08	0.79 ± 0.11
	*AUC*		0.87 ± 0.07	0.84 ± 0.09	0.91 ± 0.05	0.80 ± 0.10
**IPMN**	*Precision*		0.33 ± 0.42	0.83 ± 0.16	0.94 ± 0.05	0.80 ± 0.19
	*Recall*		0.11 ± 0.13	0.43 ± 0.18	0.85 ± 0.06	0.48 ± 0.27
	*AUC*			0.64 ±0.17	0.84 ± 0.10	0.73 ± 0.13
**MCN**	*Precision*			0.68 ±0.23	0.88 ± 0.03	0.70 ± 0.23
	*Recall*			0.92 ± 0.16	1.00 ± 0.00	0.98 ± 0.04
	*AUC*				0.74 ± 0.10	0.74 ± 0.13
**PanIN**	*Precision*				0.78 ± 0.06	0.63 ± 0.24
	*Recall*				0.99 ± 0.01	0.85 ± 0.15
	*AUC*					0.84 ± 0.04
**PDAC**	*Precision*					0.63 ± 0.19
	*Recall*					0.51 ± 0.20

The AUC, precision and recall scores on the held-out test set for each pairwise classifier is listed here. The proposed CGAT framework results in significant improvement in pairwise classification accuracy over the MH indices based classifier.

**Table 3** shows the confusion matrix of a multi-class classifier derived from bootstrapping multiple pairwise classifiers. The diagonal entries indicate the number of correctly classified instances on the held-out test set. The label for each row indicates the ‘true’ class, whereas the off-diagonal entries indicate the ‘predicted’ class. It can be observed that of the 12 examples labeled as CP, the model correctly identifies 11 of them. Similarly, PDAC is correctly identified on 28 out of 30 instances. The performance on other classes is not as noteworthy, primarily due to lack of both quality and quantity of the available data.

**Table 3:** The confusion matrix for the multi-class CGAT classifier for the six different pancreatic diseases.

	CP	IPMN	MCN	PanIN	PDAC	IPMN-associated PDAC
CP	11	1	0	0	0	0
IPMN	6	8	0	0	4	0
MCN	2	0	1	0	2	0
PanIN	2	2	0	2	2	0
PDAC	1	1	0	0	28	0
IPMN-associated PDAC	1	2	0	0	2	3

The weighted (class-normalized) precision, recall and *F*_1_-scores for the confusion matrix were obtained as 0.73, 0.65 and 0.62, respectively.

As mentioned earlier, the Morisita Horn dissimilarity indices were also computed for each image in the cohort to assess the performance of our classifier. A set of decision tree pairwise classifier models were trained for classifying index values for many two pairs of diseases. **Table 4** shows the AUC, precision and recall performances obtained from these models. As is evident, we find that the model performs poorly in comparison to the CGAT model for all disease pairs, with the pairs of PDAC and IPMN, PDAC and PanIN, IPMN-associated PDAC and CP, and IPMN-associated PDAC and PDAC performing barely better than a random classifier with AUC of 0.5. This claim is further strengthened by a strong overlap observed in the index values between all six groups, as depicted in **Supplementary Figure S1**. The AUC values of less than 0.5 observed for the other disease pairs can be explained by the significant class imbalance observed in this dataset.

**Table 4:** Classification metrics for the 15 pairwise decision-tree classifiers based on the Morisita-Horn index values from every point pattern set from each disease group.

Groups	Scores	IPMN	MCN	PanIN	PDAC	IPMN-associated PDAC
	*AUC*	0.41 ± 0.02	0.37 ± 0.06	0.35 ± 0.09	0.45 ± 0.06	0.53 ± 0.08
**CP**	*Precision*	0.31 ± 0.02	0.60 ± 0.05	0.48 ± 0.08	0.19 ± 0.12	0.64 ± 0.08
	*Recall*	0.38 ± 0.10	0.50 ± 0.16	0.50 ± 0.10	0.17 ± 0.10	0.57 ± 0.07
	*AUC*		0.41 ± 0.04	0.41 ± 0.04	0.54 ± 0.04	0.40 ± 0.03
**IPMN**	*Precision*		0.74 ± 0.02	0.64 ± 0.03	0.45 ± 0.06	0.63 ± 0.03
	*Recall*		0.79 ± 0.13	0.69 ± 0.08	0.33 ± 0.07	0.65 ± 0.12
	*AUC*			0.30 ± 0.04	0.53 ± 0.06	0.45 ± 0.04
**MCN**	*Precision*			0.18 ± 0.04	0.16 ± 0.11	0.13 ± 0.18
	*Recall*			0.24 ± 0.09	0.20 ± 0.14	0.08 ± 0.11
	*AUC*				0.41 ± 0.05	0.31 ± 0.06
**PanIN**	*Precision*				0.04 ± 0.09	0.30 ± 0.06
	*Recall*				0.02 ± 0.05	0.30 ± 0.11
	*AUC*					0.52 ± 0.04
**PDAC**	*Precision*					0.80 ± 0.02
	*Recall*					0.91 ± 0.03

The AUC, precision and recall scores each pairwise classifier is listed here. It can be observed that classification accuracy of several pairwise classifiers built upon single MH index per image is significantly poor.

## 4 Discussion

Despite the advances in pathology imaging paradigms and computational methods, challenges in discrimination between pathologies with similarities still continues to persist. This is specifically seen in the case of pancreatic cancers and their precursor conditions (36). Additionally, surveying the immune landscape and the arrangement and clustering of cells in the disease microenvironment may be crucial in deciphering disease progression and in the development of effective therapeutic regimens. Though spatial methods like the Morisita-Horn dissimilarity index have been used to quantify spatial relationships, they are usually limited to two distinct cell phenotypes. To the best of our knowledge, there has not been an attempted to use this metric to capture the relationships fully in multiplexed data sets (such as mIF imaging data or transcriptomics data) where multi-way phenotype relationships have the potential to be assessed. In this study, we proposed and applied a graph attention-based classification method on a cohort of imaging data from different pancreatic disorders. Rather than utilize single cell features that usually call for extensive feature set creation, our network relies on cell identity and relative locations as inputs for the classification paradigm.

On the application of our framework to the study data, we observe that the *k*-NN based classification paradigm was able to perform significantly better than the single-number Morisita-Horn indices across all pairwise comparisons. Specifically, we observed that our classifier is able to distinguish between CP and PDAC in a significantly better manner, as opposed to the MH Index. From a clinical perspective, this discrimination is highly relevant, as misdiagnosis of these two diseases with frequently similar pathological appearances may lead to either a missed diagnosis of a severe carcinoma, or repeated biopsies due to the high cancer risk of patients with previous history of CP (37). Similar performance improvements were observed between PDAC and its precursor and co-occurring conditions like MCN and PanIN. This alludes to a nuance in the neighborhood relationships between the three cell phenotypes utilized in this study, which may have been missed during visual observation. Furthermore, our frame can offer hypothesis generation tools for biologists to interrogate the tumor micro-environment.

**Figure 3:**
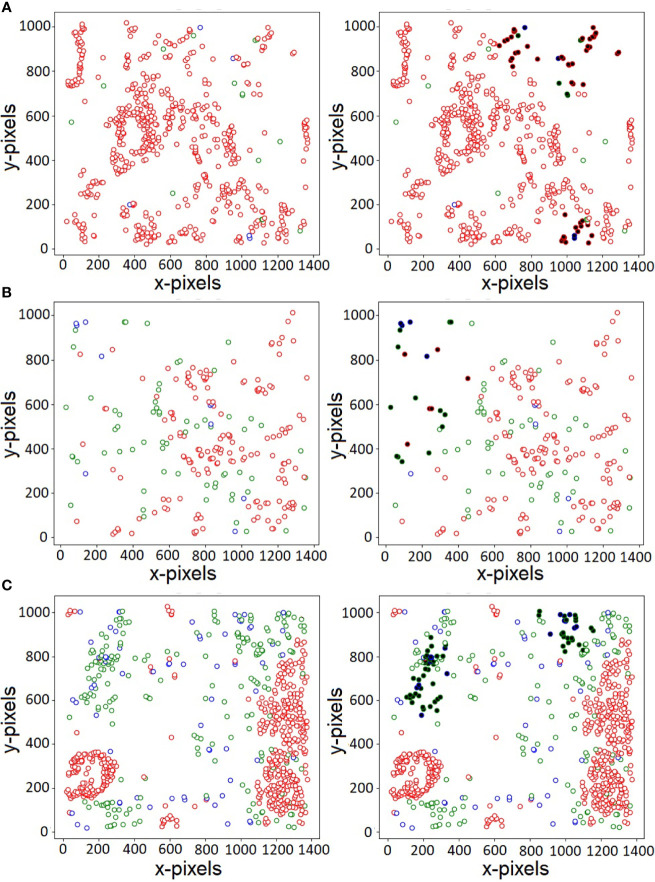
Visualization of nuclei with large attention weights for three sample PDAC histopathology images **(A–C)**. Red, green and blue colors indicate the three types of labels - Epithelial, CTL and Treg, respectively. The cells with large attention weights likely belong to the interface of two or more cell types, indicating patterns that govern the various class association.

Of the various cells present in the disease micro-environment, cytotoxic lymphocytes (CTLs) have a functionally significant presence, and play an active role in regulating anti-tumor response, specifically in PDAC (38). Of the various subtypes, the anti-tumor CTLs and immunoregulatory Tregs play a large role in this, with opposing effects on immune mediation and disease prognosis. It has been known that T-regulatory cells play an active role in the immunosuppressive environment present in PDAC, and thus have a greater probability to co-localize and have an inhibitory effect on the more immunoreactive CTLs present in the environment (39). One of the advantages of our proposed CGAT-network is its ability to highlight *important* nuclei through its self-attention mechanism. The self-attention mechanism helps the model to focus only on specific parts of the input, while learning to ignore unimportant details. Attention mechanisms are not only known to boost model’s predictive performance, they can also assist in better visualization of the features that are likely responsible for the predictive capabilities of the model. **Figure 3** shows the original histopathological images (on the left) and the corresponding attention-visualized images (on the right) for three representative images from the PDAC class. The attention weights are computed as described in (2), and the cells with attention scores in the 90^th^-percentile are highlighted in bold. As observed in **Figure 3**, these cells are observed to be those in closer proximity to cells of other phenotypes, rather than their own. Additionally, the regions with the highest attention weights also overlaps with regions of higher Treg density (marked in blue). This further lends credence that the presence of Tregs influences the spatial positions, and in turn the functional effect of cytotoxic lymphocytes on epithelial cells in the cancer environment. Further identification and exploration of the spatial arrangement of other associated cell phenotypes at these interaction interfaces holds the key in quantifying the state of the cancer micro-environment in PDAC. This, in turn can potentially lead to more robust methods not only to prevent misdiagnosis, but also is key in earlier identification of the disease for more effective treatment paradigms to be delivered.

To reiterate, CGAT’s ingenuity lies in the utilization of only the nuclei class labels for the construction of the *k*-NN based cell graph used as input for the classification model. This alleviates the need for extensive feature engineering, reducing reliance on the computation of secondary graph summary metrics such as centrality measures. The ability of the attention network to delineate nuclei, and in turn explicitly point to regions influencing classification is a tool that can be leveraged in the identification of novel cell-cell relationships that have been not been previously deemed as influential. This is in sharp contrast to previously applied neural network based methods, which essentially function as a black box and do not completely give context into the decision making process for classification. The identification of these cell clusters can help in the identification of functionally influential cell-cell arrangement of the same phenotype as well as other phenotypes.

We wanted to closely analyze the regions of “attention” pointed out to us by the CGAT framework, and attempt to interpret them from a physiological context. We chose to examine the results from the CP-PDAC classifier, as that had been our best performer, and was of great interest from a biological perspective. For this purpose, we first segmented out the cells lying within the top 50^th^ percentile of attention weights obtained from the CP-PDAC binary classifier, as identified by our framework. This was used as input for the Giotto framework, and the results are mentioned in **Tables 2** and **3** in the **Supplementary Material**. The results from the high attention weight regions point to a few trends. Firstly, we observed an enrichment of Tregs around other Tregs in PDAC with no such phenomenon in CP. This is consistent with known literature stating that factors present in the cancer microenvironment are driving the location-specific polarization of these regulatory cells (40). In contrast, a depletion in neighborhood relationships was observed between Tregs and epithelial cells in the attention regions in PDAC, which goes against current domain knowledge, and potentially suggests that another cell type present in the microenvironment might be influencing Treg polarization. Additionally, it was also observed that there was a depletion in neighborhood proximity between epithelial cells and CTLs in CP when compared to a random neighborhood model, which can be explained by the global inflammatory nature of the disease (41, 42). Though the strength of the relationships were not strong enough, the significance of these contrasting relationships observed between the high attention cells identified by the classifier warrants further exploration in future studies. This would further help us in characterizing the state of the disease micro-environment in a spatially informed manner (43). The availability of this information can also help in deeper analysis of phenotype relationships already postulated in previous literature for related diseases.

While the proposed work uses only three different cell types for disease class prediction, the CGAT framework is quite general and can accommodate any number of cell types by appropriately modifying the maximum size of the dictionary of embeddings in the embedding layer. In an ideal scenario, it would be preferred to have more than just three cell markers. This is reflected in our analysis (see **Table 1 in the Supplementary Material**) with just two markers - “Tumor” and “Immune”, i.e., both the CTL and Treg markers are masked as a single “Immune” marker. We show that even with just these two markers, the reduction in performance of the proposed CGAT architecture is not significant, indicating that even in the absence of multiple cell markers, CGAT is capable of distinguishing different pancreatic diseases better than the Morisita-Horn index-based approach involving three different cell markers. Due to the generalizability of the framework, its application can be extended to other omics data as well, where spatial information is available. A limiting factor in this study is the disproportionate number of image data sets available from each cohort, biasing a model towards the class with the larger membership. Application of this framework on a more balanced data set would be the next step to diminish this effect, and potentially gain a even higher classification accuracy with better metrics. It is to be noted that even with the disparity in the number of samples per diseases, our model was still able to perform appreciatively well in discerning between any two pairs of diseases.

In conclusion, this proposed and implemented cell-graph based method for the classification of mIF image-derived point patterns obtained from six different cohorts of pancreatic diseases. Instead of focusing on expensive feature engineering, we work with cell-graphs consisting of only one feature per node. With only 3 phenotypes of cells segmented out in each image, this method was able to display excellent classification metrics between all possible pairs of the diseases. An extension of this workflow on a more balanced dataset with a richer amount of cell phenotypic information available would be warranted.

## Data Availability Statement

The datasets presented in this article are not readily available because of restrictions on licensing for use by the University of Michigan School of Medicine. The data sets may be available from the corresponding author on request, with appropriate permissions from the institution. Requests to access the datasets should be directed to AR, ukarvind@umich.edu.

## Ethics Statement

The studies involving human participants were reviewed and approved by University of Michigan Institutional Review Board. The patients/participants provided their written informed consent to participate in this study.

## Author Contributions

Methodology: MB, SK, MO, AR. Acquisition of data: TF, SK Writing and reviewing of the manuscript: MB, SK, MO, TF, AR. Supervision: AR. All authors contributed to the article and approved the submitted version.

## Funding

AR, SK and MO were supported by CCSG Bioinformatics Shared Resource 5 P30 CA046592, a gift from Agilent technologies, a Research Scholar Grant from the American Cancer Society (RSG-16-005-01), the NCI Grant R37-CA214955, and The University of Michigan (U-M) startup institutional research funds. AR and SK were also partially supported by Precision health Investigator award from U-M Precision Health to AR along with LR and MS. MO was supported by the Advanced Proteome Informatics of Cancer Training Grant (T32 CA140044). None of the funders were involved in the study design, collection, analysis, and interpretation of data, the writing of this article or the decision to submit it for publication.

## Conflict of Interest

AR has a consulting agreement with Voxel analytics LLC and consults for Genophyll, LLC. MB is currently employed with the Division of Data and Decision Sciences, Tata Consultancy Services, India.

The remaining authors declare that the research was conducted in the absence of any commercial or financial relationships that could be construed as a potential conflict of interest.

The funders were not involved in the study design, collection, analysis, and interpretation of data, the writing of this article or the decision to submit it for publication.

## Publisher’s Note

All claims expressed in this article are solely those of the authors and do not necessarily represent those of their affiliated organizations, or those of the publisher, the editors and the reviewers. Any product that may be evaluated in this article, or claim that may be made by its manufacturer, is not guaranteed or endorsed by the publisher.
