# Supplementary material for: CGAT: Cell Graph ATtention Network for Grading of Pancreatic Disease Histology Images
Source: Front Immunol. 2021 Sep 29;12:727610. doi: 10.3389/fimmu.2021.727610 (PMC8522581; doi:10.3389/fimmu.2021.727610)
Supplement: Supplementary file 1 [file DataSheet_1.pdf]

Supplementary Material for "CGAT: Cell Graph ATtention Network for Grading of Pancreatic Disease Histology Images"

Mayank Baranwal [1,2*], Santhoshi Krishnan [3,4], Morgan Oneka [4], Timothy Frankel [5] and Arvind Rao [3,4,6,7,8,*]

[1] *Division of Data & Decision Sciences, Tata Consultancy Services Research, Mumbai, Maharashtra, 400607, India*
[2] *Department of Systems and Control Engineering, Indian Institute of Technology, Bombay, Maharashtra, 400076, India*
[3] *Department of Electrical & Computer Engineering, Rice University, Houston, TX,USA*
[4] *Department of Computational Medicine and Bioinformatics, University of Michigan, Ann Arbor, MI, USA*
[5] *Department of Surgery, University of Michigan, Ann Arbor, MI, USA*
[6] *Department of Biostatistics, University of Michigan, Ann Arbor, MI, USA*
[7] *Department of Radiation Oncology, University of Michigan, Ann Arbor, MI, USA*
[8] *Department of Biomedical Engineering, University of Michigan, Ann Arbor, MI, USA*

Correspondence*:
Dr. Mayank Baranwal
baranwal.mayank@tcs.com

Dr. Arvind Rao
ukarvind@umich.edu

1 THE MORISITA-HORN DISSIMILARITY INDEX

The Morisita-horn similarity index is a measure derived from, and used the field of ecology, where it was used to study predator-prey relationships among species in a given environment. This measure has been adapted for use in quantifying the co-localization of any two pairs of cell phenotypes in the tumor micro-environment, and among other cancer types, has been shown to be predictive of breast cancer [(1, 2)]. The value of the indices range from 0 to 1, with 0 indicating no overlap(or co-localization from the perspective of its application) between the two phenotypes of cells, and 1 indicating perfect overlap of cells of both phenotypes in each of the observed "tessellations" in a given image. Formally, the index is mathematically defined as,

$$MH_{(c_1,c_2)} = \frac{2\sum_k \frac{c_{k1}}{\sum c_{i1}} \frac{c_{k2}}{\sum c_{i2}}}{\sum_k (\frac{c_{k1}}{\sum c_{i1}})^2 + \sum_k (\frac{c_{k2}}{\sum c_{i2}})^2} \tag{1}$$

where, c_1 and c_2 are the two cell phenotype populations of interest, and k being the number of unique species/phenotypes present. In the context of our paper, we set the quadrant to be a 250 x 250 microns square, for computing the index value, as was proposed in the original paper [(1)] The index computations and associated analyses were implemented in R (R Core Team (2013)).

2 SUPPLEMENTARY TABLES AND FIGURES

2.1 Figures

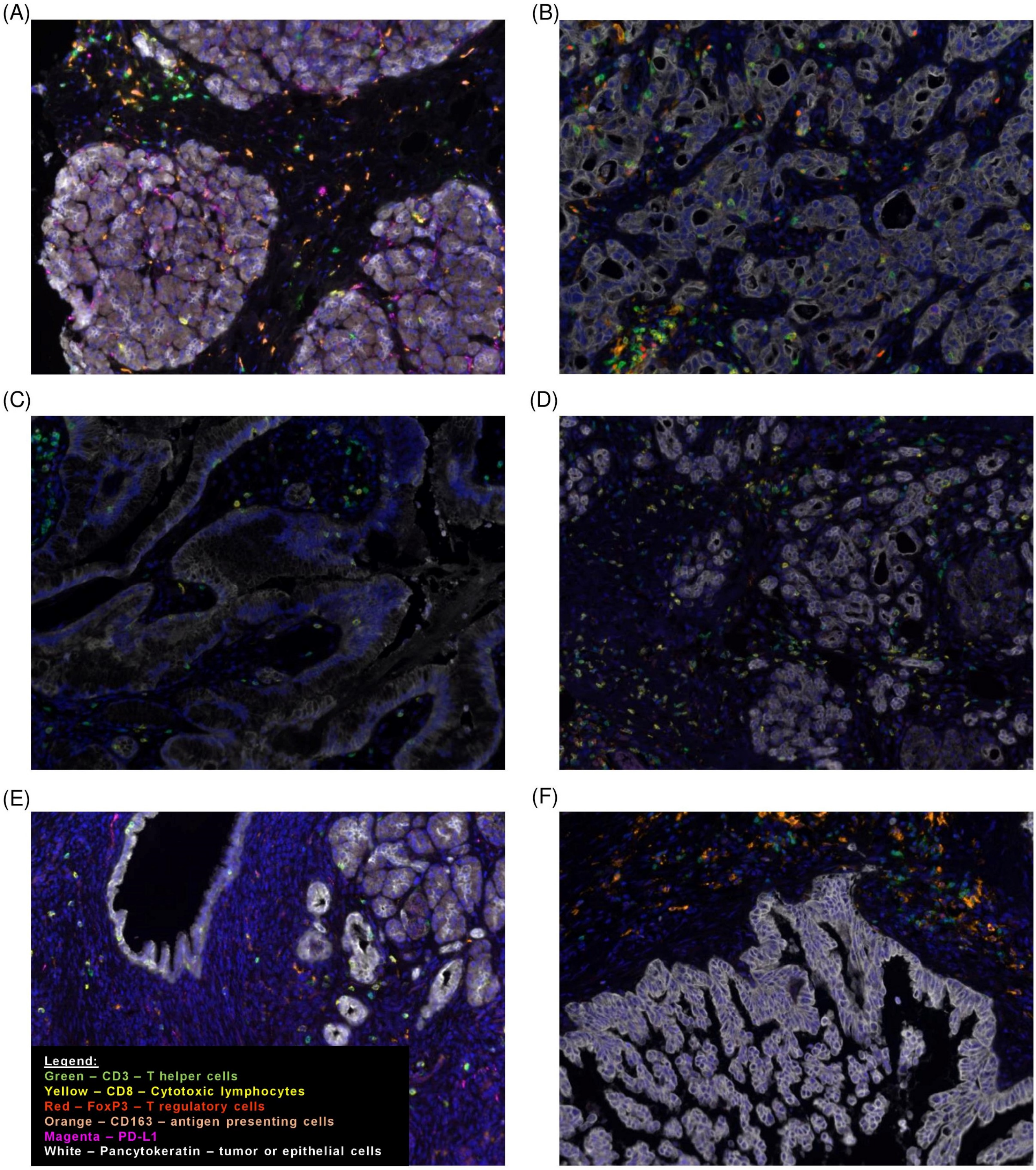

Figure 1. A representative subset of multiplexed immunofluorescence (mIF) image from our study cohort, with (A)-(F) representing sample images from Chronic Pancreatitis, PDAC, IPMN, PanIN, MCN, and IPMN-associated PDAC, respectively. Image courtesy Dr. Timothy L. Frankel, University of Michigan Department of Surgery.

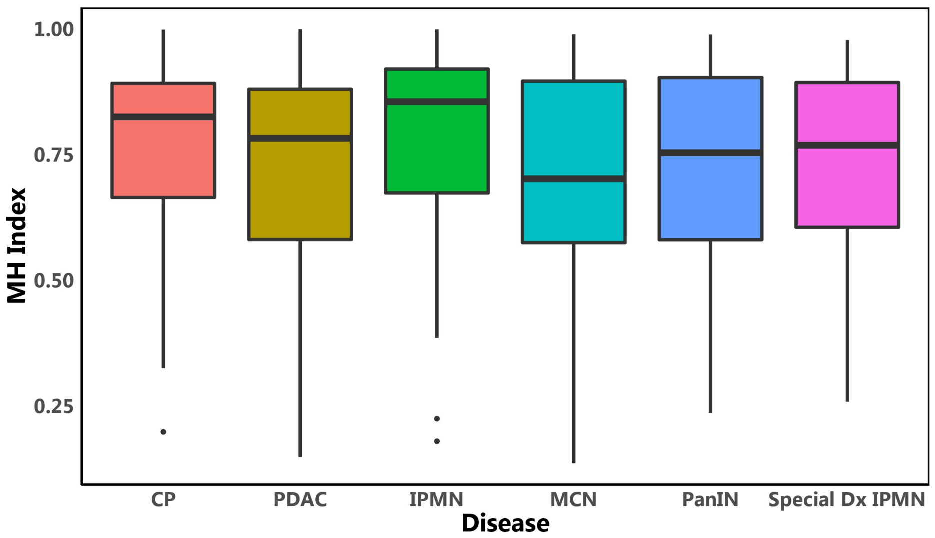

Figure 2. Box-plots depicting the spread of the Morisita-Horn Index across all 6 pancreatic disease groups. As it can be seen, there is a considerable overlap in the range of values from each group, thus strengthening the claim that the Morisita-Horn index does not effectively capture the difference in cellular arrangement, especially in a limited data cohort such as the one used in our study.

2.2 Tables

Groups	Scores	IPMN	MCN	PanIN	PDAC	IPMN-associated PDAC
**CP**	*AUC*	0.57±0.01	0.70±0.03	0.56±0.06	0.77±0.00	0.63±0.01
	Precision	0.68±0.27	0.75±0.00	0.61±0.02	1.00±0.00	0.64±0.03
	Recall	0.30±0.27	1.00±0.00	1.00±0.00	0.00±0.00	0.93±0.10
**IPMN**	*AUC*		0.87±0.07	0.56±0.00	0.64±0.00	0.63±0.05
	Precision		0.61±0.04	0.69±0.00	1.00±0.00	0.69±0.00
	Recall		0.78±0.00	0.96±0.09	0.00±0.00	1.00±0.00
**MCN**	*AUC*			0.70±0.02	0.50±0.09	0.53±0.05
	Precision			1.00±0.00	1.00±0.00	1.00±0.00
	Recall			0.16±0.08	0.00±0.00	0.20±0.00
**PanIN**	*AUC*				0.78±0.01	0.73±0.02
	Precision				1.00±0.00	0.68±0.09
	Recall				0.00±0.00	0.82±0.13
**PDAC**	*AUC*					0.67±0.01
	Precision					0.79±0.00
	Recall					1.00±0.00

Table 1. Classification metrics for the 15 pairwise CGAT classifiers from every point pattern set from each disease group with just two cell-markers (“Tumor” and “Immune”). The AUC, precision and recall scores on the held-out test set for each pairwise classifier is listed here.

Cell 1	Cell 2	Enrichment score	P-value (Enrichment)	P-value (Depletion)
Treg	Treg	-0.911	1	0.42
Treg	Tumor	-0.046	0.987	0.014
Treg	CTL	0.281	0.008	0.992
Tumor	Tumor	0.002	0	1
CTL	CTL	0.078	0	1
CTL	Tumor	-0.013	1	0

Table 2. Results from the Giotto framework for Chronic Pancreatitis.

Cell 1	Cell 2	Enrichment score	P-value (Enrichment)	P-value (Depletion)
Treg	Treg	0.146	0	1
Treg	Tumor	-0.032	1	0
Treg	CTL	0.012	0.095	0.908
Tumor	Tumor	0.008	0	1
CTL	CTL	0.005	0.207	0.795
CTL	Tumor	-0.005	0.92	0.08

Table 3. Results from the Giotto framework for PDAC.

REFERENCES

1 .Maley CC, Koelble K, Natrajan R, Aktipis A, Yuan Y. An ecological measure of immune-cancer colocalization as a prognostic factor for breast cancer. *Breast Cancer Research* **17** (2015). doi:10.1186/s13058-015-0638-4.

2 .Wolda H. Similarity indices, sample size and diversity. *Oecologia* **50** (1981) 296–302. doi:10.1007/bf00344966.
